# Phase II clinical and endocrine study of Anandron (RU-23908) in advanced post-menopausal breast cancer.

**DOI:** 10.1038/bjc.1991.170

**Published:** 1991-05

**Authors:** M. J. Millward, B. M. Cantwell, M. Dowsett, J. Carmichael, A. L. Harris

**Affiliations:** University Department of Clinical Oncology, Newcastle General Hospital, Newcastle-Upon-Tyne, UK.


Br. J. Cancer (1991), 63, 763-764 © Macmillan Press Ltd., 1991

SHORT COMMUNICATION

### Phase II clinical and endocrine study of Anandron (RU-23908) in advanced post-menopausal breast cancer

M.J. Millward[1], B.M.J. Cantwell[1], M.Dowsett[2], J. Carmichael[3] & A.L. Harris[3]

[1]*University Department of Clinical Oncology, Newcastle General Hospital, Newcastle-Upon-Tyne NE4 6BE;* [2]*Department of Academic Biochemistry, The Royal Marsden Hospital, Fulham Road, London SW3 6JJ; and* [3]*ICRF Clinical Oncology Unit, Churchill Hospital, Oxford OX3 7LJ, UK.*

Androgen receptors are present in 30-50% of primary breast carcinomas (Bretani *et al.*, 1986; Miller *et al.*, 1985; Bryan *et al.*, 1984) and their presence is correlated with oestrogen receptor positivity (Bretani *et al.*, 1986; Miller *et al.*, 1985), age in post-menopausal women (Bretani *et al.*, 1986) and response to endocrine therapy, but not to chemotherapy (Bryan *et al.*, 1984). The administration of pharmacologic doses of androgen (fluoxymestrone) has shown anti-tumour activity in women refractory to tamoxifen (Manni *et al.*, 1981) but is associated with virilising side effects. Since both high doses of oestrogens as well as anti-oestrogens such as tamoxifen are clinically active in post-menopausal patients with breast cancer, an anti-androgen might be expected to have similar therapeutic efficacy to androgens, but without virilising side effects. Anandron (RU-23908) (5,5-dimethyl-3(4-nitro-3(trifluoromethyl)phenyl)2, 4 imidazolidinedione) is a nonsteroidal anti-androgen that competitively inhibits the effects of testosterone at the receptor level (Moguilewsky *et al.*, 1987). It is considered to be a pure anti-androgen since it has no androgenic, oestrogenic, anti-oestrogenic, progestomimetic or antiprogesterone activity and does not bind to the mineralocorticoid or glucocorticoid receptor. After oral administration it is rapidly and completely absorbed with a slow plasma half-life allowing once daily dosage when steady state levels are achieved after 2 weeks (Moguilewsky *et al.*, 1987). Anandron in doses of 100-300 mg $day^{-1}$ has been used in men with metastatic prostate cancer either after or in combination with medical or surgical castration and has been well tolerated (Moguilewsky *et al.*, 1987; Kuhn *et al.*, 1989). Here we report a phase II clinical study using Anandron in post-menopausal women with advanced breast cancer and the resulting endocrine effects. As far as we are aware this is the first use of Anandron in breast cancer.

Postmenopausal (including post therapeutic oophorectomy) women with measurable advanced breast cancer progressing on standard therapy and a life expectancy of 3 months were eligible for entry on study. Anandron was given in a dose of 100 mg once daily continuously. Patients were assessed clinically at weekly intervals for the first 4 weeks then 4 weekly until disease progression. Hormone assays were performed for oestradiol, follicle stimulating hormone (FSH), luteinising hormone (LH) and sex hormone binding globulin (SHBG) in ten patients and 5-alpha dihydrotestosterone (DHT) in four patients using previously described methods (Ferguson *et al.*, 1982; Dowsett *et al.*, 1985; Dowsett *et al.*, 1987; Dowsett *et al.*, 1989).

Fifteen patients were enrolled on study. One patient who did not attend for any follow up visits and was of indeterminate menopausal status was excluded from analysis. The remaining patients are all evaluable for response and toxicity. Patient details are shown in Table I. Three patients had had a previous therapeutic oophorectomy. The median number of previous endocrine therapies was three (range one to four) and ten patients had previously received chemotherapy, principally single agent mitoxantrone, for advanced disease. Oestrogen receptor status of the primary tumour was known for only two patients both of whom were positive. The lack of oestrogen receptor measurements is attributable to the use of non-surgical methods of treatment for primary disease in most patients because of its advanced nature or the patients' advanced age.

**Table I** Patient details

| | |
|---|---|
| *No. of patients* | 14 |
| *Age* mean (year) | 70 |
| range | 46-87 |
| *Previous endocrine therapy* | |
| tamoxifen | 14 pts |
| aminoglutethimide | 12 pts |
| progestogen | 7 pts |
| oophorectomy | 3 pts |
| LHRH agonist | 2 pts |
| ketoconazole | 2 pts |
| *Response to previous endocrine therapy* | |
| yes | 7 pts |
| no | 5 pts |
| not assessable | 2 pts |
| *Disease sites* | |
| local recurrence | 7 pts |
| lymph node | 2 pts |
| cutaneous | 4 pts |
| lung | 6 pts |
| bone | 6 pts |
| pleura | 2 pts |
| contralateral primary | 1 pt |
| liver | 1 pt |
| *Dominant disease site* | |
| loco-regional | 9 pts |
| visceral | 4 pts |
| bone | 1 pt |

No objective responses were seen. Two patients had disease stabilisation; one for 26 weeks (lung-previous response to tamoxifen, but progression on aminoglutethimide/hydrocortisone and megestrol acetate) and one for 20 weeks (breast, bone, lung-previous progression on tamoxifen). The latter patient has had stable disease on aminoglutethimide/hydrocortisone for 42+ weeks following progression on Anandron. The other patient with stable disease did not receive further therapy. Of the 11 patients who had progressive disease on Anandron four had subsequent alternative hormone therapy with one brief partial response to megestrol acetate and five had subsequent chemotherapy with no objective responses.

There was no toxicity definitely related to Anandron. One patient stopped treatment after 4 weeks because of subjective swelling and altered sensation on one side of the tongue and suspicion of an allergic reaction but was found to have progression of bone metastases in the base of the skull. Another patient died at home following a chest infection

Correspondence: B.M.J. Cantwell, University Department of Clinical Oncology, Newcastle General Hospital, Westgate Road, Newcastle-Upon-Tyne NE4 6BE, UK.
Received 15 November 1990; and in revised form 3 January 1991.

while taking Anandron, but an autopsy was not performed and therefore the syndrome of Anandron-related interstitial pneumonitis which has been reported in 2% of patients treated with Anandron could not be excluded. Anandron has been reported to induce difficulty with visual light/dark adaptation in 24% of men treated for prostatic carcinoma (Moguilewsky *et al.*, 1987) but despite specific questioning no patient in this study reported this side-effect.

The results of the endocrine measurements are shown in Table II. No significant changes were seen although there was a rise in SHBG that approached significance ($P = 0.09$).

This study did not demonstrate any objective responses using Anandron, however two patients had stabilisation of previously progressive disease for $\geqslant 20$ weeks. Howell *et al.* (Howell *et al.*, 1988) have shown that this duration of disease stability with endocrine therapy for advanced breast cancer confers the same progression free and overall survival advantage as partial response and therefore Anandron may have some anti-tumour activity in advanced breast cancer. Although our patients were mainly elderly with loco-regional disease they had received a median of three previous endocrine therapies and the majority previous chemotherapy and it is possible that more definite anti-tumour activity would be seen in less heavily pre-treated patients. A National Cancer Institute of Canada Clinical Trials Group study (Perrault *et al.*, 1988) using a closely similar anti-androgen (flutamide) found one partial response and five stable disease in 29 evaluable women with advanced breast cancer, but premenopausal and known oestrogen receptor negative patients were included. Unlike Anandron, flutamide was associated with troublesome gastro-intestinal toxicity.

**Table II** Effect of Anandron on mean (± s.e.m.) oestradiol, FSH, LH, 5-alpha DHT and SHBG in postmenopausal women

| | *Baseline* | *Week 4* | *P value (2 tail)* |
|---|---|---|---|
| Estradiol (pmol $l^{-1}$) | 64.4 ± 24.9 | 59.0 ± 19.0 | 0.55 |
| FSH (IU $l^{-1}$) | 29.2 ± 6.8 | 26.2 ± 7.1 | 0.22 |
| LH (IU $l^{-1}$) | 25.3 ± 6.4 | 26.4 ± 7.0 | 0.61 |
| 5-alpha DHT (nmol $l^{-1}$) | 0.16 ± 0.02 | 0.14 ± 0.02 | 0.59 |
| SHBG (nmol $l^{-1}$) | 53.1 ± 11.1 | 72.4 ± 13.6 | 0.09 |

There were no significant changes in the endocrine parameters suggesting that Anandron has no major peripheral endocrine effects in postmenopausal women. There was a tendency for elevation of SHBG which is consistent with the known action of exogenous androgens in suppressing plasma levels of SHBG. The lack of a major measurable endocrine effect does not however exclude a pharmacologic effect through interaction with tumour androgen receptors. Higher doses of Anandron have been used in men with prostatic cancer (Moguilewsky *et al.*, 1987) and it is possible that a different dose may have different effects in post-menopausal women. Further clinical and endocrine evaluation of Anandron is justified in minimally pre-treated, potentially hormone sensitive breast cancer given its low incidence of toxicity. Where possible patient's tumour tissue should be assayed for androgen receptor content.

### References

BRETANI, M.M., FRANCO, E.L., OSHIMA, C.T.F. & PACHECO, M.M. (1986). Androgen, estrogen and progesterone receptor levels in malignant and benign breast tumours: a multivariate analysis approach. *Int. J. Cancer*, **38**, 637.

BRYAN, R.M., MERCER, R.J., BENNET, M.S., RENNIE, G.C., LIE, T.H. & MORGAN, F.J. (1984). Androgen receptors in breast cancer. *Cancer*, **54**, 2436.

DOWSETT, M., ATTREE, S.L., VIRDEE, S.S. & JEFFCOATE, S.L. (1985). Oestrogen related changes in sex hormone binding globulin levels during normal and gonadotropin-stimulated menstrual cycles. *Clin. Endocrinol.*, **23**, 303.

DOWSETT, M., GOSS, P.E., POWLES, T.J. & 4 others (1987). Use of the aromatase inhibitor 4-hydroxyandrostenedione in postmenopausal breast cancer: optimization of therapeutic dose and route. *Cancer Res.*, **47**, 1957.

DOWSETT, M., CUNNINGHAM, D.C., STEIN, R.C. & 4 others (1989). Dose-related endocrine effects and pharmacokinetics of oral and intramuscular 4-hydroxyandrostenedione in postmenopausal breast cancer patients. *Cancer Res.*, **49**, 1306.

FERGUSON, K., HAYES, M. & JEFFCOATE, S.L. (1982). A standardized multicentre procedure for plasma gonadotropin radioimmunoassay. *Ann. Clin. Biochem.*, **19**, 358.

HOWELL, A., MACINTOSH, J., JONES, M., REDFORD, J., WAGSTAFF, J. & SELLWOOD, R.A. (1988). The definition of the 'no change' category in patients treated with endocrine therapy and chemotherapy for advanced carcinoma of the breast. *Eur. J. Cancer Clin. Oncol.*, **24**, 1567.

KUHN, J.-M., BILLEBAUD, T., NAVRATIL, H. & 9 others (1989). Prevention of the transient adverse effects of a gonadotropin-releasing hormone analogue (Buserelin) in metastatic prostatic carcinoma by administration of an antiandrogen (Nilutamide). *N. Engl. J. Med.*, **321**, 413.

MANNI, A., ARAFAH, B.M. & PEARSON, O. (1981). Androgen-induced remissions after antiestrogen and hypophysectomy in stage IV breast cancer. *Cancer*, **48**, 2507.

MILLER, W.R., TELFORD, J., DIXON, J.M. & HAWKINS, R.A. (1985). Androgen receptor activity in human breast cancer and its relationship with oestrogen and progesterone receptor activity. *Eur. J. Cancer Clin. Oncol.*, **21**, 539.

MOGUILEWSKY, M., BERTAGNA, C. & HUTCHER, M. (1987). Pharmacologic and clinical studies of the antiandrogen Anandron. *J. Steroid. Biochem.*, **27**, 871.

PERRAULT, D.J., LOGAN, D.M., STEWART, D.J., BRAMWELL, V.H.C., PATERSON, A.H.G. & EISENHAUSER, E.A. (1988). Phase II study of flutamide in patients with metastatic breast cancer. A National Cancer Institute of Canada Clinical Trials Group study. *Invest. New Drugs*, **6**, 207.


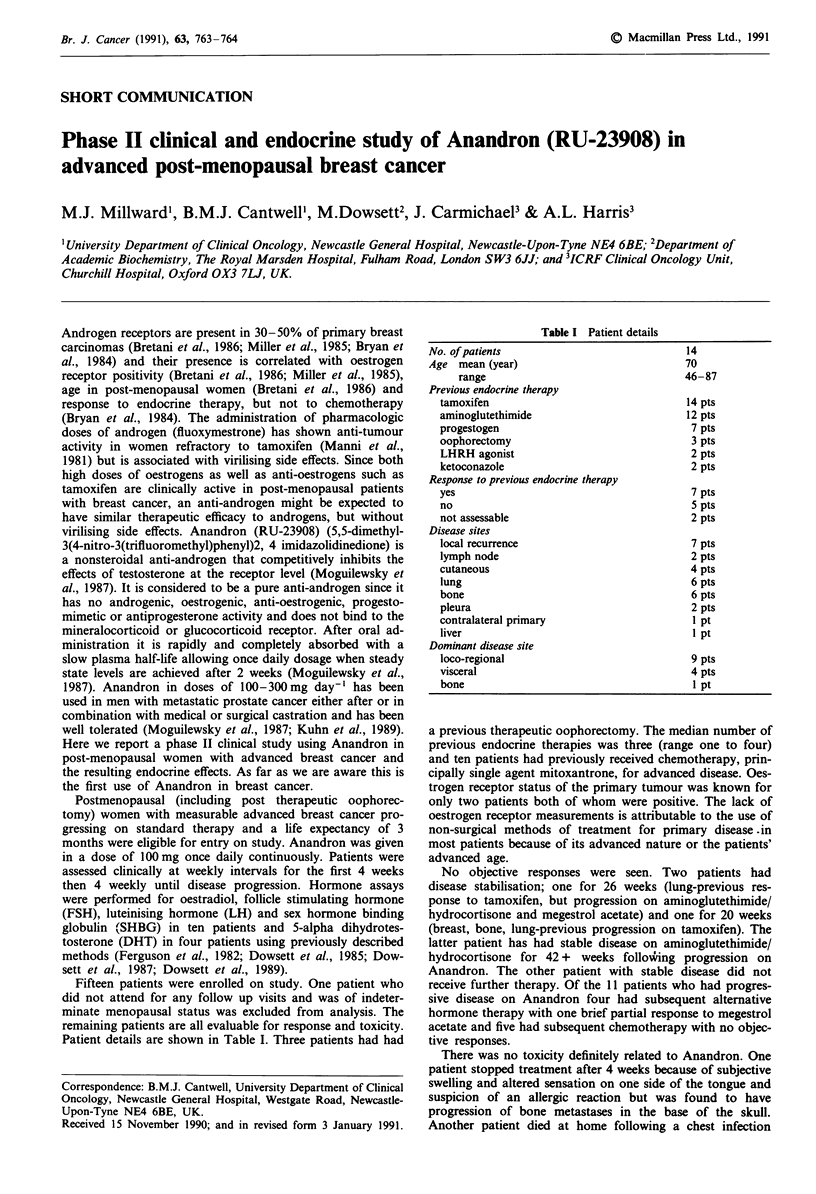

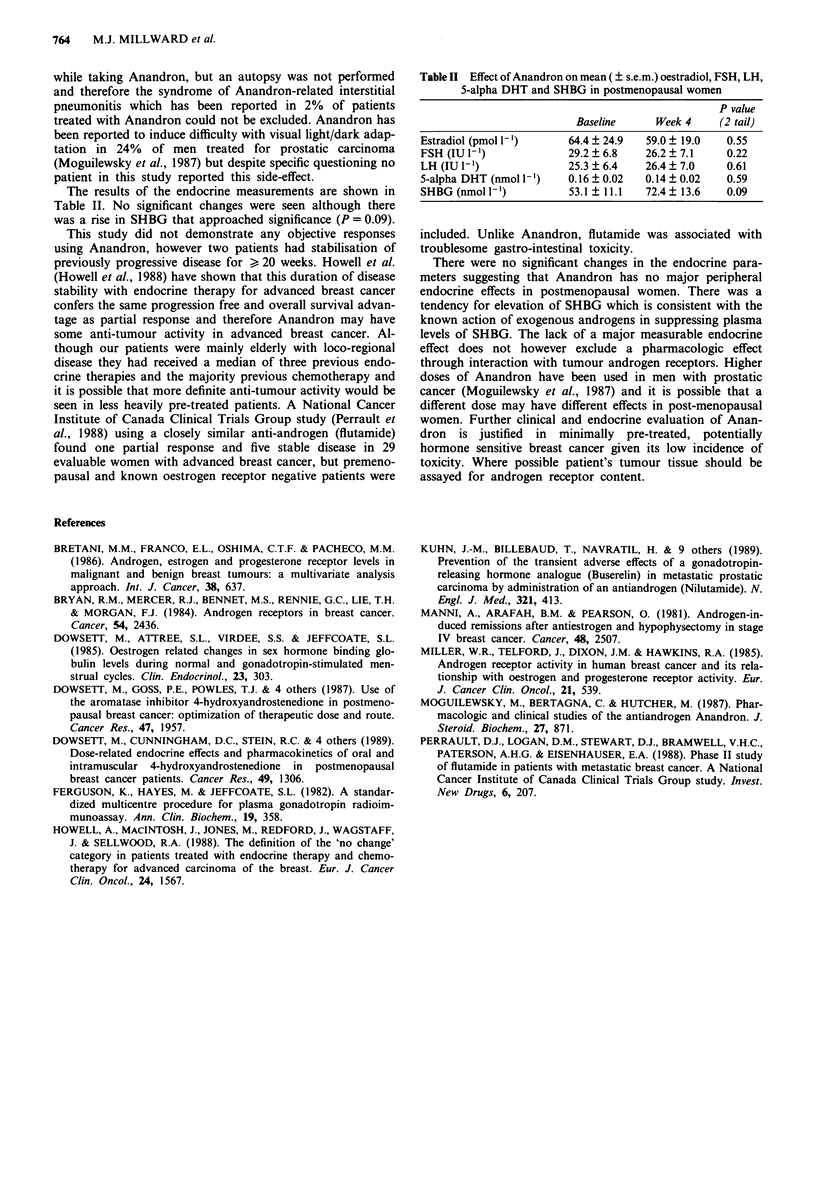


## References

[OCR_00249] Brentani M. M., Franco E. L., Oshima C. T., Pacheco M. M. (1986). Androgen, estrogen, and progesterone receptor levels in malignant and benign breast tumors: a multivariate analysis approach.. Int J Cancer.

[OCR_00255] Bryan R. M., Mercer R. J., Bennett R. C., Rennie G. C., Lie T. H., Morgan F. J. (1984). Androgen receptors in breast cancer.. Cancer.

[OCR_00260] Dowsett M., Attree S. L., Virdee S. S., Jeffcoate S. L. (1985). Oestrogen-related changes in sex hormone binding globulin levels during normal and gonadotrophin-stimulated menstrual cycles.. Clin Endocrinol (Oxf).

[OCR_00272] Dowsett M., Cunningham D. C., Stein R. C., Evans S., Dehennin L., Hedley A., Coombes R. C. (1989). Dose-related endocrine effects and pharmacokinetics of oral and intramuscular 4-hydroxyandrostenedione in postmenopausal breast cancer patients.. Cancer Res.

[OCR_00266] Dowsett M., Goss P. E., Powles T. J., Hutchinson G., Brodie A. M., Jeffcoate S. L., Coombes R. C. (1987). Use of the aromatase inhibitor 4-hydroxyandrostenedione in postmenopausal breast cancer: optimization of therapeutic dose and route.. Cancer Res.

[OCR_00278] Ferguson K. M., Hayes M., Jeffcoate S. L. (1982). A standardised multicentre procedure for plasma gonadotrophin radioimmunoassay.. Ann Clin Biochem.

[OCR_00283] Howell A., Mackintosh J., Jones M., Redford J., Wagstaff J., Sellwood R. A. (1988). The definition of the 'no change' category in patients treated with endocrine therapy and chemotherapy for advanced carcinoma of the breast.. Eur J Cancer Clin Oncol.

[OCR_00290] Kuhn J. M., Billebaud T., Navratil H., Moulonguet A., Fiet J., Grise P., Louis J. F., Costa P., Husson J. M., Dahan R. (1989). Prevention of the transient adverse effects of a gonadotropin-releasing hormone analogue (buserelin) in metastatic prostatic carcinoma by administration of an antiandrogen (nilutamide).. N Engl J Med.

[OCR_00297] Manni A., Arafah B. M., Pearson O. H. (1981). Androgen-induced remissions after antiestrogen and hypophysectomy in stage IV breast cancer.. Cancer.

[OCR_00302] Miller W. R., Telford J., Dixon J. M., Hawkins R. A. (1985). Androgen receptor activity in human breast cancer and its relationship with oestrogen and progestogen receptor activity.. Eur J Cancer Clin Oncol.

[OCR_00308] Moguilewsky M., Bertagna C., Hucher M. (1987). Pharmacological and clinical studies of the antiandrogen Anandron.. J Steroid Biochem.

[OCR_00313] Perrault D. J., Logan D. M., Stewart D. J., Bramwell V. H., Paterson A. H., Eisenhauer E. A. (1988). Phase II study of flutamide in patients with metastatic breast cancer. A National Cancer Institute of Canada Clinical Trials Group study.. Invest New Drugs.

